# Chemical profile of essential oil from *Pimenta racemosa* leaves, antioxidant potential, and its enzyme inhibitory properties

**DOI:** 10.1038/s41598-025-15542-3

**Published:** 2025-08-21

**Authors:** Omayma A. Eldahshan, Nilofar Nilofar, Gokhan Zengin, Heba A. S. El-Nashar

**Affiliations:** 1 Department of Pharmacognosy, Faculty of Pharmacy, Ain Shams University, Abbassia, Cairo, 11566 Egypt; 2 Department of Biology, Science Faculty, Selcuk University, Campus, Konya, Turkey; 3 Department of Pharmacy, Botanic Garden “Giardino dei Semplici”, Università degli Studi “Gabriele d’Annunzio”, via dei Vestini 31, 66100 Chieti, Italy

**Keywords:** Acetylcholinesterase, Antioxidant, Eugenol, GC-MS, *Pimenta racemosa*, Tyrosinase, Plant sciences, Medical research

## Abstract

*Pimenta racemosa* var. recemosa (Mill.) J. W. Moore is an aromatic plant belonging to the family Myrtaceae, native to Venezuela, Puerto Rico, and Jamaica and well-known for its traditional and medicinal uses. Our study was designated to explore the chemical composition of essential oil isolated from *P. racemosa* leaves growing in Egypt *via* Gas Chromatography/Mass Spectrometry (GC-MS) analysis, alongside investigation of its antioxidant properties and enzyme inhibitory activities against acetylcholinesterase (AChE), butyrylcholinesterase (BChE), tyrosinase, *α*-glucosidase, and *α*-amylase. The GC-MS analysis of the leaf oil revealed the presence of fourteen compounds (99.76%), predominated by eugenol (70.87%), *β*-myrcene (12.88%) and D-limonene (8.35%). The oil demonstrated the highest antioxidant capability in ferric ion-reducing antioxidant power (FRAP; 1506.62 mg TE/g), followed by 2,2’-azino-bis(3-ethylbenzothiazoline-6-sulphonic acid (ABTS; 1346.85 mg TE/g), 1,1-diphenyl-2-picrylhydrazyl (DPPH; 1032.83 mg TE/g) and cupric-reducing antioxidant capacity (CUPRAC; 1001.03 mg TE/g). Further, it showed a metal chelating ability (MCA) of 25.63 mg EDTAE/, and phosphomolybdenum (PBD) activity of 209.59 mmol TE/g. The oil displayed significant AChE and BChE inhibitory activities, with values of 1.96 mg GALAE/g and 1.42 mg GALAE/g, respectively. Additionally, it exhibited a moderate level of tyrosinase inhibitory activity (38.83 mg KAE/g) and a significantly higher α-glucosidase inhibitory activity (2.38 mmol ACAE/g) than α-amylase (0.08 mmol ACAE/g). Consequently, the leaf oil of *Pimenta racemosa* could be used as adjuvant therapy for management of oxidative stress-related conditions and chronic diseases such as Alzheimer’s, diabetes mellitus, and skin pigmentation. However, further toxicological, *in vivo* and clinical studies are recommended.

**Supplementary Information:**

The online version contains supplementary material available at 10.1038/s41598-025-15542-3.

## Introduction

In contemporary times, medicinal plants represent an essential therapy for some people especially in Western countries due to the undesirable effects of synthetic chemical drugs and the substantial financial benefits^1,2^. However, despite the difficulty of estimating the exact commercial value of plants and plant extracts, it is clear that folk medicine-related industries grow by more than 4% annually^3,4^. The standing of medicinal plants and non-conventional plants is attributed to the high multiplicity of bioactive components, like flavonoids, alkaloids, lignans, triterpenoids, essential oils, carotenoids, tocopherols, and vitamins^5,6^. Some of these bioactive molecules demonstrate remarkable antioxidant properties which are essentially searched for their valuable impact on various chronic diseases, like Alzheimer’s disease (AD), skin wrinkles, pigmentation, obesity, diabetes mellitus, and cancer^7–9^.

Natural products including essential oils have been recognized *via* scientific reported literature for their activities against AD. Tumeric (*Curcuma longa)*^10^, guava (*Psidium guajava*)^11^, and bitter orange (C*itrus aurantium*)^12^ showed potential against acetylcholinesterase and butyrylcholinesterase. Tyrosinase is a key enzyme in melanogenesis. The antityrosinase activity of many plants has also been studied for the development of functional cosmetics because the inhibition of this enzyme can attenuate melanin synthesis to induce a whitening effect. Several medicinal plants have been reported for tyrosinase inhibitory effects such as Milk thistle (*Silybum marianum)*, lemongrass (*Cymbopogon citrates)*, and licorice (*Glycyrrhiza glabra*)^13^.

Recently, there has been renewed interest in functional food products and plant-based therapies that modify the physiological properties for the management of diabetes mellitus and aging symptoms^14,15^. One of the targeted antidiabetic approaches is to inhibit carbohydrate-metabolizing enzymes like α-glucosidase and α-amylase^3^. Thus, hyperglycemia can be managed in the type-II diabetic patients and borderline patients^3,16^. Arrays of previous in vitro studies have shown the potential role of several plants as α-amylase and α-glucosidase inhibitors such as cranberry (*Vaccinium macrocarpon*)^17^, black plum (*Syzygium cumini*)^18^, turmeric (*Curcuma longa*)^19^, Black pepper (*Piper nigrum*)^20^, soybean (*Glycine max*)^21^ Fenugreek (*Trigonella foenum-graecum* ) and turmeric (*Curcuma longa*)^22^. Consequently, the plant-derived α-glucosidase and α-amylase inhibitors provide an attractive strategy to restrain postprandial hyperglycemia.

The *Pimenta* genus is one of the evergreen flowering trees that incorporates about fifteen species and belongs to the family Myrtaceae^23,24^. *Pimenta* plants are distributed in West Indies, Central America and Jamaica^25^. In traditional medicine, different *Pimenta* species were employed for treatment of common cold, viral infections, bronchitis, dental pain, muscle aches, rheumatic pains, and arthritis^26^. Moreover, *Pimenta* essential oils exert antimicrobial, antiseptic, anesthetic, and analgesic effects^25^. From the phytochemical aspect, the isolated essential oils of *Pimenta* plants are characterized by an abundance of eugenol, methyl eugenol, *β*-caryophyllene, and myrcene^27^. Whereas limonene, 1,8-cineole, terpinolene, *β*-selinene, and methyl eugenol were reported to be the predominant components in some studies^28^. *P. racemosa* (Mill.) J. W. Moore is one of the most characteristic aromatic plants of this genus, due to its high essential oil content and diverse medicinal targets^29^.*P. racemosa* is native to Venezuela, Puerto Rico, and Jamaica in the Caribbean^30^. It is characterized by the richness of essential oil which is commonly named “bay oil” or “Myrcia oil”^30^. Traditionally, *P. racemosa* was utilized for different diseases in the Dominican Republic of the Caribbean basin; the leaf oil is used for rheumatism and toothache due to its anti-inflammatory and analgesic effects^31^. The infusion of the bark is used against hypertension^30^. The leaf essential oil was reported to possess antimicrobial effects against *Staphylococcus aureus*, *Escherichia coli*, *Pseudomonas aeruginosa*, *Candida albicans*, *Aspergillus niger*, *Penicillium verrucosum* and *Cladosporium cladosporioides*^32^. Several biological studies have demonstrated its anti-cancer^33^, anti-inflammatory^34^, nematicidal activity^35^, and insecticidal activities^36^.

*In* *vitro* antidiabetic assays are important to determine the activity of plant extract or compounds hence saving time, cost, and effort^37^. Interestingly, no previous data have been reported on the antioxidant or enzyme inhibition data of *P. racemosa* leaf oil. Thus, the current study was designated to explore the chemical composition of essential oil isolated from *P. racemosa* leaves growing in Egypt *via* GC-MS chemical analysis, alongside investigation of its antioxidant property and enzyme inhibitory activity against acetylcholinesterase (AChE), butyrylcholinesterase (BChE), tyrosinase, α-glucosidase, and α-amylase.

## Materials and methods

### Plant material and oil isolation

*P. racemosa* (Myrtaceae) were collected as fresh leaves in March 2023 from El-Zohrya Garden (30°02′45″N 31°13′28″E), Zamalek, Cairo Governorate, Egypt. The collection of plant material was permitted and established in compliance with the national guidelines. A voucher specimen under code PHG-P-PR-473 was put at the Herbarium of the Department of Pharmacognosy, at the Faculty of Pharmacy, Ain Shams University, Cairo, Egypt. The leaves were accurately authenticated by Mrs. Treize Labib, a taxonomy expert at El-Orman Botanical Garden, Giza-Egypt. About 1 kg of the fresh leaves were finely cut, and hydrodistilled using a Clevenger apparatus for a period of 4 h. Afterwards, the essential oil isolation was accomplished and stored in a sealed glass tube at -5 °C waiting for GC-MS analysis and further biological exploration.

### Chemicals

The chemicals were purchased from Sigma-Aldrich (Darmstadt, Germany). They were: 2,2’-azino-bis(3-ethylbenzothiazoline-6-sulphonic acid (ABTS), 1,1-diphenyl-2-picrylhydrazyl (DPPH), electric eel acetylcholinesterase (AChE) (type-VI-S, EC 3.1.1.7), horse serum butyrylcholinesterase (BChE) (EC 3.1.1.8), galantamine, acetylthiocholine iodide (ATChI), butyrylthiocholine chloride (BTChI) 5,5-dithio-bis(2-nitrobenzoic) acid (DTNB), tyrosinase (EC1.14.18.1, mushroom), glucosidase (EC. 3.2.1.20, from *Saccharomyces cerevisiae*), amylase (EC. 3.2.1.1, from the porcine pancreas), sodium molybdate, hydrochloric acid, sodium hydroxide, trolox, ethylenediaminetetraacetate (EDTA), neocuproine, cupric chloride, ammonium acetate, ferric chloride, 2,4,6-Tris(2-pyridyl)-s-triazine (TPTZ), ammonium molybdate, ferrozine, ferrous sulphate hexahydrate, kojic acid, and acarbose. All chemicals were of analytical grade.

### Gas chromatography/mass spectrometry (GC-MS) analysis

At the pharmacognosy department of Ain Shams University, Cairo, Egypt, the isolated oil was analyzed with a Shimadzu GCMS-QP 2010 (Kyoto, Japan) and a TRACE GC Ultra Gas Chromatograph (THERMO Scientific Corp., USA), coupled to a thermo-mass detector as previously reported^38–40^. The column specifications (Restek, USA) include a TG-5MS capillary with an internal diameter of 30 m x 0.25 mm i.d. and film thickness of 0.25 μm, directly attached to a quadrupole mass spectrometer (SSQ7000; Thermo-Finnigan). The carrier gas is helium with a constant flow rate of 1.0 mL/min. The sample is diluted with a ratio 1% v/v, the injected volume = 1 µL, and a split ratio of 1:15. Initially, the oven temperature of 80 °C was maintained for 2 min (isothermal), and then 300 °C was reached by raising 5.0 °C/min and retaining it for 5 min (programmed). The temperatures of the injector and detector were set at 280 °C. Using an electron ionization (EI) mode of 70 eV, a scanned spectrum at m/z 35–500 was used to obtain mass spectra of the ions: interface temperature = 280 °C, ion source temperature = 200 °C. A peak area normalization technique was used to determine the relative proportions of the hexane extract constituents.

#### Percentage peak area method

The percentage peak area method uses the area of the target component peak as a proportion of the total area of all detected peaks to analyze quantity. This method is used to determine an approximate concentration of a sample mixture or changes in the concentration of a known sample mixture.

#### GC-MS identification of chemical components of the essential oil

To tentatively identify oil components, the GC-FID and GC-MS spectra were used, and fragmentation patterns, mass numbers, and Kovats retention indices were compared with those published by Wily, NIST, and literature sources^18,41–46^. Under the same conditions, homologous series of *n*-alkanes (C8-C28) were injected, and retention indices were calculated. In this method, peak areas were calculated as a ratio of the peak areas of each compound to the area of the entire FID chromatogram (100%).

### Antioxidant and enzyme inhibitory assays

The essential oil was subjected to six spectrophotometric tests to determine its antioxidant potential. The essential oil solution was carefully formulated in ethanol at a concentration of 2 mg/mL. All chemicals were freshly prepared, with special attention to keeping the enzymes and their corresponding substrates in an ice bath for upcoming. We prepared an ethanolic solution of the essential oil, and then transferred it to the reaction medium. To assess the antioxidants’ aptitude to neutralize free radicals, 2,2′-azino-bis(3-ethylbenzothiazoline-6-sulfonic acid (ABTS) and 1,1-diphenyl-2-picrylhydrazyl (DPPH) assays were employed^47^. Also, the reduction capabilities of isolated essential oil were evaluated through ferric ion-reducing antioxidant power (FRAP) and cupric-reducing antioxidant capacity (CUPRAC) tests^47^. Furthermore, phosphomolybdenum (PBD) and ferrozine assays were used to measure the antioxidant activity and metal-chelating capability of the essential oil, respectively^47,48^. Apart from metal chelating ability (MCA), each of these assays was valued using a Trolox standard (mg TE/g). As for MCA, its comparison was estimated according to the EDTA equivalent per gram of essential oil (mg EDTAE/g). All procedures are detailed in our previous works^47,48^. To explore the inhibitory effects of the tested oil on various enzymes, we applied acetylcholinesterase (AChE), butyrylcholinesterase (BChE), tyrosinase, and amylase, and glucosidase and the experimental procedures were described previously in our earlier work^48,49^. As a measure of AChE and BChE inhibition, we measured the inhibition using milligram galantamine equivalents per gm of essential oil (mg GALAE/g), the inhibition of tyrosinase expressed as mg kojic acid equivalents per gm essential oil (mg KAE/g), and the inhibition of amylase expressed in milligrams of acarbose equivalents per gm essential oil (mg ACAE/g). The measurements of these antioxidant and enzyme assays were carried out using a microplate reader ((Thermo Multiskan GO, Thermo, United States). Each assay was carried out with three technical replicates. The details of each essay were shown in the supplementary file. Further, the calibration curves of all used standards are illustrated in Table S1.

## Results and discussion

### GC-MS analysis of leaf essential oil from *P. racemosa*

**Fig. 1:**
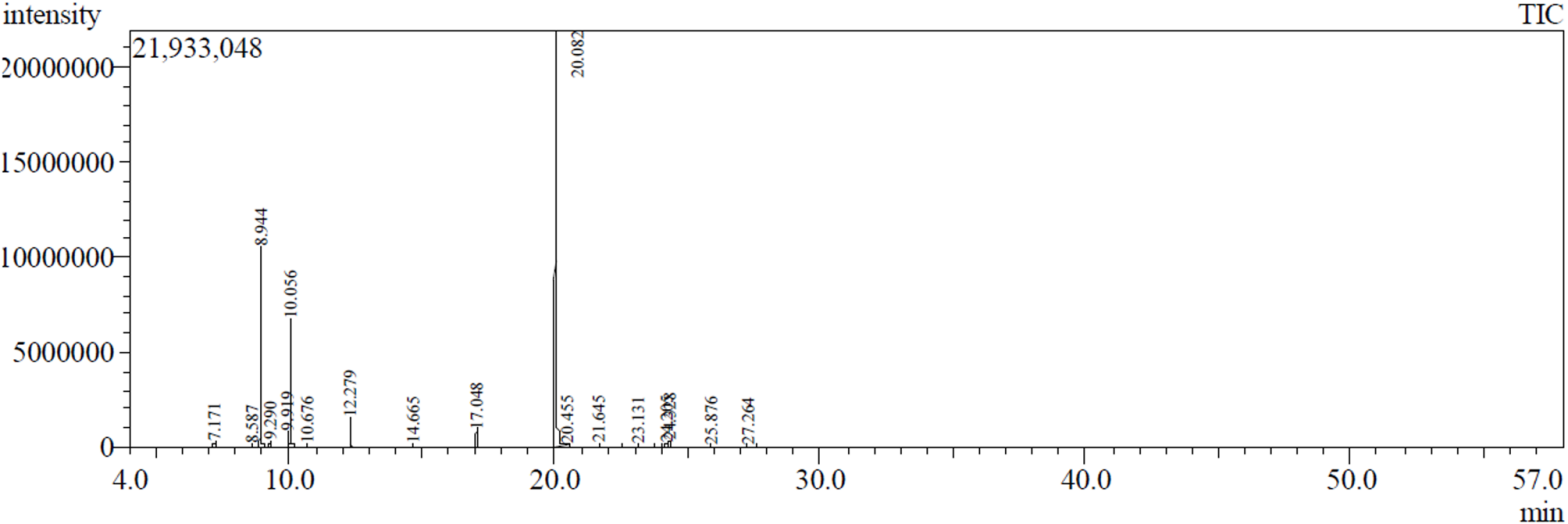
GC-MS chromatogram of the leaf essential oil isolated from *P. racemosa* grown in Egypt.

**Fig. 2:**
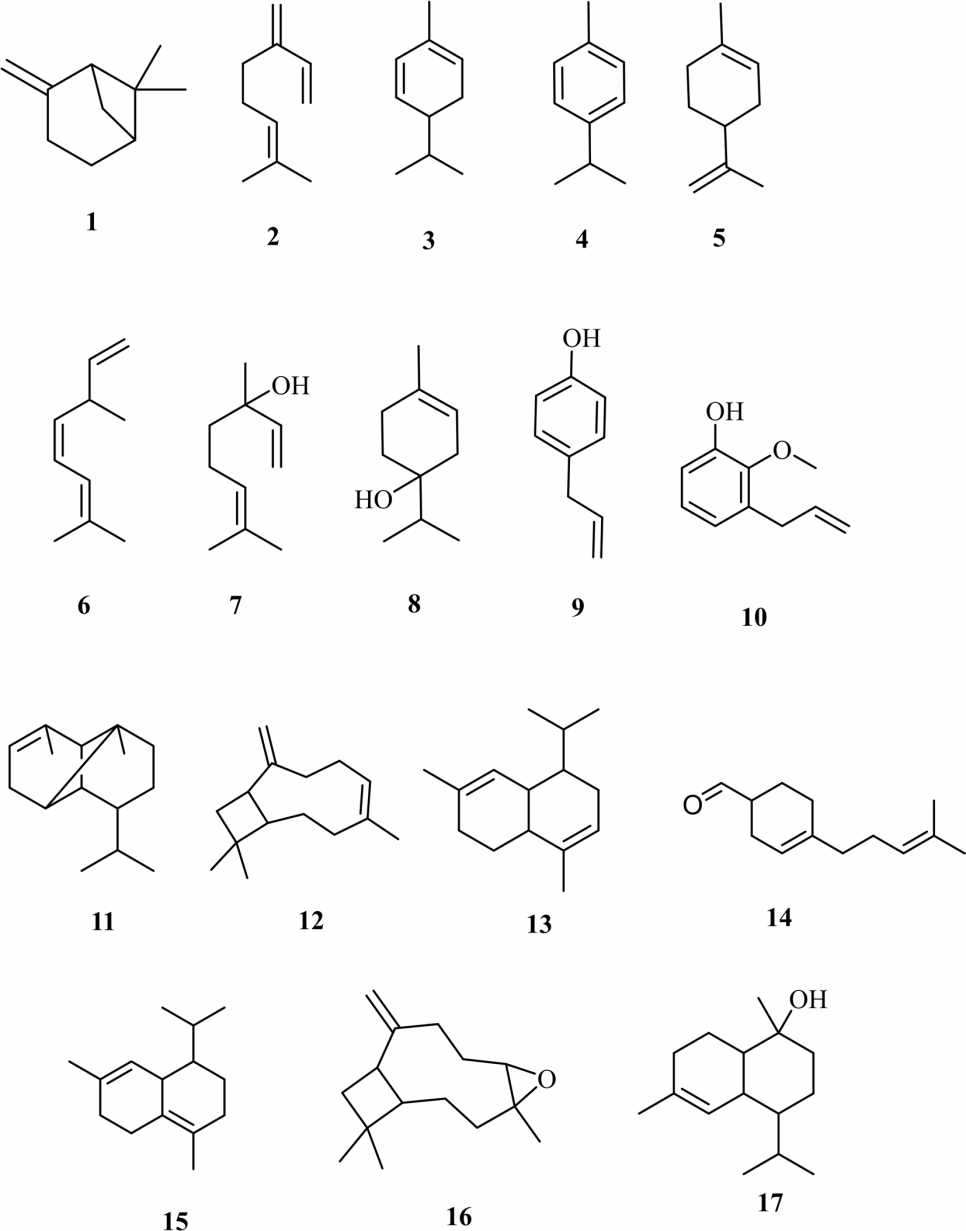
The structures of the volatile components detected in the essential oil of *P. racemosa* leaves grown in Egypt [1: *α*-Pinene, 2: *β*-Myrcene, 3: *α*-Phellandrene, 4: *p*-Cymene, 5: D-Limonene, 6: (*E*)-*β*-Ocimene, 7: *β*-Linalool, 8: Terpinen-4-ol, 9: Chavicol, 10: Eugenol, 11: α-Copaene, 12: (*E*)-*β*-Caryophyllene, 13: α-Murolene, 14: 3-Cyclohexene-1-carboxaldehyde, 15: 4-(4-methyl-3-pentenyl)-, 16: δ-Cadinene, (*E*)-*β*-Caryophyllene oxide, 17: τ-Cadinol].

The GC-MS analysis was employed to characterize the volatile components of essential oil isolated from *P. racemosa* leaves. It revealed a depiction of seventeen compounds constituting about 99.76% of the total oil components as illustrated in Table 1 and Fig 1. The Phenylpropanoids are the chief class of oil components representing 72.35%, among them eugenol (70.87%) and chavicol (1.48%). Hydrocarbon monoterpenes represent the second major class of oil components with a total percentage of 23.41% predominated by *β*-myrcene (12.88%), D-limonene (8.35%), and *p*-cymene (1.12%). The oxygenated monoterpenes accounted for about 2.33% of total oil including *β*-linalool (2.14%) and terpinene-4-ol (0.19%). Hydrocarbon sesquiterpene showed a minor composition percentage of oil (0.32%) represented by (*E*)-*β*-caryophyllene oxide (0.16%) and *τ*-cadinol (0.16%). The chemical structures of the identified major oil components are illustrated in Fig. 2. Certain Egyptian studies reported a predominance of monoterpene hydrocarbons (64.40%) predominated by *β*-myrcene (39.60%) and eugenol (31.00%) in *P. racemosa* leaf oils^25^. While the Cuban type of *P. racemosa* leaf oil demonstrated a high content of 1,8-cineole (14.69%)^50^. In Benin, the leaf essential oil was quantified with eugenol (45.20%), myrcene (25.10%), chavicol (7.10%), and limonene (3.0%) as major compounds^30^. Unlike that, another study in Santo Domingo demonstrated the presence of *α*-terpineol acetate (27%), *α*-terpineol (20%), and methoxy eugenol (12.6%)^31^. It is assumed that these differences in the chemical composition of the above-mentioned essential oils can be ascribed to differences in the cultivation environment, geographical resources, climate, genotype, and collection time^51^. Few literatures about the chemical nature of the essential oil of *P. racemosa* var. racemose were reported which matched our result. Contreras-Moreno et al., and Tucker et al., revealed the presence of eugenol as the major compound in the same chemotype (60.4–82.9%; 44.41–68.93% respectively)^52,53^. The main oil components previously identified in *P. racemosa*, their percentage, and the location are illustrated in Table 2.

**Table 1:** GC-MS chemical composition (%) of the essential oil from *P. racemosa* grown in egypt.

No.	Compound name ^a^	Retention time (t_*R*_)	Molecular formula	RI		Peak area (%)	Method of identification	Ref.
				Experimental	Reported			
1.	*α*-Pinene	7.17	C_10_H_16_	931	932	0.32	RI, MS	^18,54^
2.	***β*** **-Myrcene**	**8.94**	**C**_**10**_ **H**_**16**_	**992**	**992**	**12.88**	**RI**,** MS**	^4,45^
3.	*α*-Phellandrene	9.29	C_10_H_16_	1003	1005	0.54	RI, MS	^40,43^
4.	*p*-Cymene	9.92	C_10_H_14_	1024	1026	1.12	RI, MS	^46^
5.	**D-Limonene**	**10.06**	**C**_**10**_ **H**_**16**_	**1028**	**1029**	**8.35**	**RI**,** MS**	^18^
6.	(*E*)-*β*-Ocimene	10.68	C_10_H_16_	1048	1050	0.20	RI, MS	^41^
7.	*β*-Linalool	12.28	C_10_H_18_O	1100	1100	2.14	RI, MS	^18^
8.	Terpinen-4-ol	14.67	C_10_H_18_O	1178	1177	0.19	RI, MS	^45^
9.	Chavicol	17.05	C_9_H_10_O	1258	1259	1.48	RI, MS	^18^
10.	**Eugenol**	**20.08**	**C**_**10**_ **H**_**12**_ **O**_**2**_	**1366**	**1365**	**70.87**	**RI**,** MS**	^51^
11.	α-Copaene	20.46	C_15_H_24_	1379	1376	0.13	RI, MS	^11^
12.	(*E*)-*β*-Caryophyllene	21.65	C_15_H_24_	1423	1423	0.28	RI, MS	^51^
13.	α-Murolene	23.13	C_15_H_24_	1480	1481	0.15	RI, MS	^45^
14.	3-Cyclohexene-1-carboxaldehyde, 4-(4-methyl-3-pentenyl)-	24.20	C_13_H_20_O	1509	1522	0.29	RI, MS	^41,46^
15.	δ-Cadinene	24.33	C_15_H_24_	1527	1530	0.50	RI, MS	^30^
16.	(*E*)-*β*-Caryophyllene oxide	25.88	C_15_H_24_O	1590	1590	0.16	RI, MS	^18^
17.	τ-Cadinol	27.27	C_15_H_26_O	1648	1648	0.16	RI, MS	^41^
	Total identified	99.76%						
	Phenylpropanoids	72.35%						
	Hydrocarbon monoterpenes	23.41%						
	Oxygenated monoterpenes	2.33%						
	Hydrocarbon sesquiterpenes	1.06%						
	Oxygenated sesquiterpenes	0.32%						
	Others	0.29						

^**a**^ Compounds are arranged according to their elution. **RI**: Kovats retention index on TG-5MS capillary column. **MS**, identification based on mass spectral data and fragmentation profile.

**Table 2:** The main oil components previously identified in *P. racemosa*, their percentage, and the location.

Main oil components (%)	Part used	Variety	Location	Refs.
Eugenol (45.2%), myrcene (25.1%), chavicol (7.1%), limonene (3.0%), and 1,8-cineole (2.1%)	Leaves	*Pimenta racemosa* (Mill.) J. W. Moore.	Godomey, Benin	^30^
β-myrcene (39.6%), Eugenol (31.0%)	Leaves	*Pimenta racemosa* (Mill.) J. W. Moore.	Zohria Garden, Egypt	^25^
β-myrcene (42.30%), Eugenol (27.7%)	Berries			
Eugenol (65.54%)	Leaves	*Pimenta racemosa* var. racemosa	Jorhat Assam, India	^54^
*α*-Terpineol acetate (27%), α-terpineol (20%) and methoxyeugenol (12.6%).	Leaves	*menta racemosa* var. terebinthina	Santo Domingo	^31^
4-methoxy-isoeugenol (75.2%) and 4-methoxyeugenol (4.5%)		*P. racemosa* var. grisea		
Eugenol (72.9%), chavicol (7.7%), β-myrcene (9.6%), limonene (3.8%)	Leaves	*Pimenta racemosa* (Mill.) J. W. Moore.	Lucknow, North India	^55^
Eugenol (57.84%)	Leaves	*Pimenta racemosa* (Mill.) J. W. Moore.	Orman Garden, Egypt	^56^
Eugenol (59.76%)	Stems			
Eugenol (46.25%), β-myrcene (25.88%), 4-Allylphenol (10.49%)	Leaves	*Pimenta racemosa* var. racemosa	West India	^57^
1,8-Cineole (75.4%) and linalool (9.08%)	Flowers	*Pimenta racemosa* (Mill.) J. W. Moore.	Zoherya Garden, Egypt	^58^
Eugenol (34.85%), *β*-pinene (18.29%), linalool (13.64%), and limonene (11.92%)	Aerial parts (leaves and small branches)	*Pimenta racemosa* (Mill.) J. W. Moore.	Giza Zoo, Egypt	^59^

### Assessment of antioxidant activity of the essential oil isolated from *P. racemosa*

Reactive oxygen species are linked to various human diseases and aging. The elevated levels of free radicals, like reactive oxygen and nitrogen species, are central to oxidative damage in biomolecules (e.g., DNA, proteins, and lipids) and play a role in food and material degradation^60–62^. These factors are also associated with chronic diseases and aging^63^. Various assays are available to assess the antioxidant capacity of samples, and this study employs six different types including 2,2-diphenyl-1-picrylhydrazyl (DPPH), [2,2′-azinobis-(3-ethylbenzothiazoline-6-sulfonic acid)] (ABTS), cupric reducing antioxidant capacity (CUPRAC), ferric reducing ability of plasma (FRAP), and phosphomolybdenum (PBD) assay as illustrated in Table 3. The essential oil isolated from *P. racemosa* leaves demonstrated remarkable antioxidant capabilities in both anti-radical and radical scavenging assays. Specifically, it exhibited the highest FRAP activity with a reducing power of 1506.62 mg Trolox equivalent (TE)/g, followed by the ABTS scavenging test at 1346.85 mg TE/g. Additionally, the essential oil displayed 1032.83 mg TE/g DPPH scavenging activity and a reducing power of 1001.03 mg TE/g in the CUPRAC assay. These antioxidant properties can be attributed to its major phenolic component, eugenol, as well as other hydrocarbon monoterpenes constituents. Furthermore, the essential oil showed a metal chelation activity (MCA) of 25.63 mg EDTAE/, and (PBD) 209.59 mmol TE/g. These results support the use of *P. racemosa* leaf essential oil as a natural antioxidant, addressing concerns about the safety of synthetic antioxidants.

Our findings closely align with the results reported by Al-Gendy et al.^58^, indicating that the *P. racemosa* flower essential oil exhibits a remarkably high scavenging capacity of 85.96%. These findings align with prior research confirming the antioxidant properties of *P. racemosa* essential oils^64^. Phenolic compounds exert antioxidant activity via different mechanisms; transference of hydrogen atom or single electron, transition metal chelation, or sequential proton loss electron transfer^65^. The robust antioxidant activity observed in the essential oil of *P. racemosa* can be attributed to its significant eugenol content, a well-established phenolic compound renowned for its antioxidative properties, as substantiated by previous studies^66–68^. Eugenol’s effectiveness as an antioxidant is, in part, attributed to the presence of a methoxy group in the ortho position of the phenolic hydroxyl, facilitating the homolytic dissociation of the O–H bond^69^. Additionally, chavicol, another phenolic compound identified in *P. racemosa* essential oil in this study, is recognized for its antioxidant activity^70^. Notably, this essential oil also contains a substantial concentration of *β*-myrcene, which serves as a major oxidation product^71^. Moreover, it is important to mention that the synergy among various compounds, even at lower concentrations, may contribute to the overall antioxidant effect.

**Table 3:** Total antioxidant effects of the tested essential oil in different in vitro assays.

Parameter	Results
DPPH (mg TE/g)	1032.83 ± 1.69
ABTS (mg TE/g)	1346.85 ± 1.32
CUPRAC (mg TE/g)	1001.03 ± 7.07
FRAP (mg TE/g	1506.62 ± 27.74
MCA (mg EDTAE/g)	25.63 ± 1.65
PBD (mmol TE/g)	209.59 ± 1.07

Values are reported as mean ± SD of three parallel measurements. TE: Trolox equivalent, DPPH: 1,1-diphenyl-2-picrylhydrazyl, ABTS: azino-bis(3-ethylbenzothiazoline-6-sulphonic acid, CUPRAC: cupric-reducing antioxidant capacity, FRAP: ferric ion-reducing antioxidant power, MCA: metal chelating ability, EDTAE: Ethylenediaminetetraacetic acid equivalent, PBD: Phosphomolybdenum assay.

### Assessment of enzyme inhibitory activities of the essential oil isolated from ***P. racemosa***

The study of enzyme inhibition is extensively researched due to its pivotal role in comprehending enzyme mechanisms^72^ and its significance in both human and animal pharmaceuticals within the realm of pharmacological research^73,74^. For example, in the case of AD, which affects over 35 million individuals globally, inhibiting cholinesterase enzymes is a key therapeutic approach^75^. Enzyme’s acetylcholinesterase (AChE) and butyrylcholinesterase (BChE) decrease acetylcholine levels, alleviating symptoms as the disease advances. Researchers are particularly interested in selective AChE and BChE inhibitors^76^. Plant bioactive compounds hold promise in effectively targeting acetylcholine-regulating enzymes for therapeutic purposes in diseases like AD^77^. Tyrosinase, another enzyme plays a central role in melanin synthesis and enzymatic browning in various contexts, including mammalian melanogenesis and fruit/fungi processes^78^. Inhibiting tyrosinase activity has broad applications in medicine and cosmetics, addressing hyperpigmentation and serving as a focal point in this study’s investigation^79^.

In the current study, in terms of enzyme inhibitory activity, the essential oil of *P. racemosa* displayed significant AChE and BChE inhibitory activities, with values of 1.96 mg GALAE/g and 1.42 mg GALAE/g, respectively (Table 4). Additionally, the essential oil exhibited a moderate level of tyrosinase inhibitory activity, measuring 38.83 mg KAE/g (Table 4). These findings underscore the potential of this essential oil in enzyme-related applications, with notable effects on acetylcholinesterase AChE and BChE inhibition, as well as a modest influence on tyrosinase inhibition.

Diabetes mellitus is a widespread epidemic, characterized by the inhibition of enzymes like α-amylase and α-glucosidase that convert dietary starch into glucose^80^. Plant extracts have shown promise in inhibiting these enzyme activities, offering potential health benefits, and serving as a focus in this study^81^. In the present study, for the 1st time, the essential oil extracted from the *P. racemosa* demonstrated a lower level of α-amylase inhibition activity, measuring at 0.08 mmol ACAE/g, while it exhibited a significantly higher α-glucosidase inhibitory activity, with a value of 2.38 mmol ACAE/g (Table 4).

In the current investigation, the plant under study exhibited a moderate inhibitory effect on the specified enzymes, as opposed to its antioxidant properties. No research has been conducted to date that demonstrates the inhibitory effects of *P. racemosa* extract or essential oil on AChE, BChE, and tyrosinase activities. Nevertheless, previous research by Adefegha et al., and Bilgicli et al., indicated that eugenol exhibited a more pronounced inhibitory effect on both AChE and BChE activities^82]– [83^. This compound possesses a diverse array of pharmacological properties, including neuromodulatory and antioxidant characteristics. Moreover, research conducted by Varela et al. and Marongiu et al. aligns with our findings, supporting the notion that eugenol exhibits a relatively less potent tyrosinase inhibition^84]– [85^. In our present study, we observed that *P. racemosa* essential oil displays a heightened level of α-glucosidase inhibitory activity when compared to its α-amylase inhibitory potential. In contrast to our current investigation, a previous study has validated that *P. racemosa* exhibits a greater inhibitory effect on α-glucosidase in comparison to α-amylase. To illustrate, the application of 2.00 mg of *P. racemosa* leaf essential oil led to a notable 63.90% increase in α-amylase inhibitory activity and a 53.00% increase in α-glucosidase inhibitory activity^86^. Similarly, several studies confirmed the pronounced α-glucosidase inhibitory properties of eugenol^83,87]– [88^. β-Myrcene is a monoterpene hydrocarbon that exerts antioxidant activity^89^ as well as neuroprotective activity where it could inhibit the acetylcholinesterase activity in different brain regions of AlCl_3_ and D - galactose-induced Alzheimer disease mice^90^. The essential oils of citrus have been reported by Matsuura et al. (2006) to possess strong antityrosinase activity due to the presence of β-myrcene^91^. Limonene, the 3^rd^ predominant component of the *Pimenta* oil, is the most abundant compound in the peel oil of orange. The latter showed strong activity against α-amylase and α-glucosidase^92^, and anti-tyrosinase inhibitory activity. The presence of the major compounds as well as the synergistic effect with other minor compounds are responsible for the activity exhibited by the oil.

**Table 4:** The enzyme inhibitory effects of the tested essential oil.

Parameters	Results
AChE (mg GALAE/g)	1.96 ± 0.25
BChE (mg GALAE/g)	1.42 ± 0.05
Tyrosinase (mg KAE/g)	38.83 ± 2.10
α-Amylase (mmol ACAE/g)	0.08 ± 0.01
α-Glucosidase (mmol ACAE/g)	2.38 ± 0.04

Values are reported as mean ± SD of three parallel measurements. AChE: acetylcholinesterase, BChE: butyrylcholinesterase, GALAE: Galantamine equivalent; KAE: Kojic acid equivalent; ACAE: Acarbose equivalent.

## Conclusion

Our findings proved that the essential oil *of P. racemosa* possesses potent antioxidant properties as confirmed in six *in vitro* assays, suggesting that it represents a highly promising resource for the development of therapeutic molecules and health-promoting agents. Our study, for the first time, indicates that the essential oil derived from *P. racemosa* substantially showed inhibitory activities against acetylcholinesterase, butyrylcholinesterase, tyrosinase, *α*-glucosidase, and *α*-amylase enzymes. This emphasizes the potential for exploiting *P. racemosa*-based preparations or adjuvant therapies for the management of chronic diseases such as Alzheimer disease, diabetes mellitus, and skin-related applications. However, further *in vivo* studies are recommended as an important phase in screening the plant oil for physiological, pharmacological, and toxicological properties.

## Supplementary Information

Below is the link to the electronic supplementary material.

Supplementary Material 1

## Data Availability

All data are described in the article and/or supplementary file.
